# Association between Serum Uric Acid and Hypertension in a Large Cross-Section Study in a Chinese Population

**DOI:** 10.3390/jcdd9100346

**Published:** 2022-10-10

**Authors:** Yang He, Du Chen, Jing-Ping Xu, Jun Jin, Jun Wang, Cong Geng, Yong-Ming He

**Affiliations:** 1 Division of Critical Care Medicine, The First Affiliated Hospital of Soochow University, Suzhou 215006, China; 2 Division of Cardiology, The First Affiliated Hospital of Soochow University, Suzhou 215006, China

**Keywords:** serum uric acid, hypertension, Chinese population

## Abstract

Background: The association of serum uric acid (SUA) with hypertension has been well established in Caucasian populations. However, its association with hypertension in Chinese remained to be clarified. Methods: Consecutive patients, homogeneous in Chinese Han ethnicity, aged ≥18 years, abstracted from the database, admitted from 1 January 2010 to 31 December 2013, were included for potential analysis. The patients were grouped according to the presence or absence of hypertension. Unconditional logistic regression was performed to estimate the association between SUA and hypertension. Its possible interactions with risk factors on hypertension were also explored. Results: A total of 9587 patients were finally analyzed in the current study, where 5692 were with hypertension and 3895 were without hypertension. Per 100 μmol/L higher SUA concentration was associated with multivariable-adjusted odds ratios (95% CI) of 1.25 (1.08–1.22) in males, 1.10 (1.01–1.20) in females, and 1.19 (1.13–1.24) in total. On a categorical scale, when compared with the first quintile, the multivariable-adjusted odds ratios (95% CI) were 1.40 (1.20–1.64) for the 2nd quintile, 1.48 (1.27–1.74) for the 3rd quintile, 1.55 (1.32–1.82) for the 4th quintile, and 1.92 (1.63–2.26) for the 5th quintile, with a *p* for trend < 0.01. Conclusions: SUA is associated with hypertension in a dose-response manner among the Chinese hospitalized population. Management of SUA could help to the prevention and control of hypertension.

## 1. Introduction

Improved living standards result in more and more people suffering from metabolic syndrome (MetS), which is a constellation of risk factors for cardiovascular disease [1]. Both high uric acid and hypertension play important roles in MetS [1]. The pathogenesis of hypertension is complex, where cardiometabolic factors play a role [2]. One of the recently proposed risk factors for hypertension is hyperuricemia [3]. The incidence rates of high uric acid and hypertension are rising in recent years, with 5.46%–19.30% and 23.2%, respectively [4]. Recent meta-analyses showed a significant association between serum uric acid (SUA) and hypertension, independently of traditional risk factors [5,6,7]. Mendelian randomized study showed a negative relationship between hyperuricemia and hypertension [8]. The limitations of these studies include that: (i) the influence of life habits and diet on SUA was ignored; (ii) most study participants were focused on the Caucasian population [9]. However, hyperuricemia susceptible genes are racially specific: SLC22A11, SLC16A9 and SLC17A1 only exist in Caucasians [10] while RFX3 and KCNQ have only been found in Chinese [11]. In addition, the median SUA was 279.2 μmol/L in Chinese and was 329.3 μmol/L in Caucasians [9,12]. Therefore, the results from Caucasians cannot be extrapolated to Chinese; and (iii) the information on the associations of SUA with essential hypertension in Chinese has been primarily from health check-up populations [13], or from small sample sized studies [12,14]. These “looking-good” populations are often with miscellaneous comorbidities, leading to misclassification in patients with or without hypertension, and ultimately to the unreliability of the results. Therefore, the present study aimed at elucidating the association between the SUA and primary hypertension using the hospitalized subjects in a large Chinese cohort.

## 2. Materials and Methods

### 2.1. The Materials

The patient data were collected from the Department of Cardiovascular Medicine in the First Affiliated Hospital of Soochow University for the period 1 January 2010 to 31 December 2013. The Institutional Review Boards waived the need for informed consent before analysis due to its retrospective nature. The study was conducted in accordance with the Declaration of Helsinki, and the protocol was approved by the Ethics Committee of The First Affiliated Hospital of Soochow University (No. 361).

The data included lab examinations, lifestyles, and prior history. The first admission data was collected for a patient with multiple hospitalizations. The first lab results were collected for a hospitalized patient with multiple lab examinations during the hospital stay. Lifestyles were smoking and alcohol consumption.

### 2.2. The Patients

**Figure 1:**
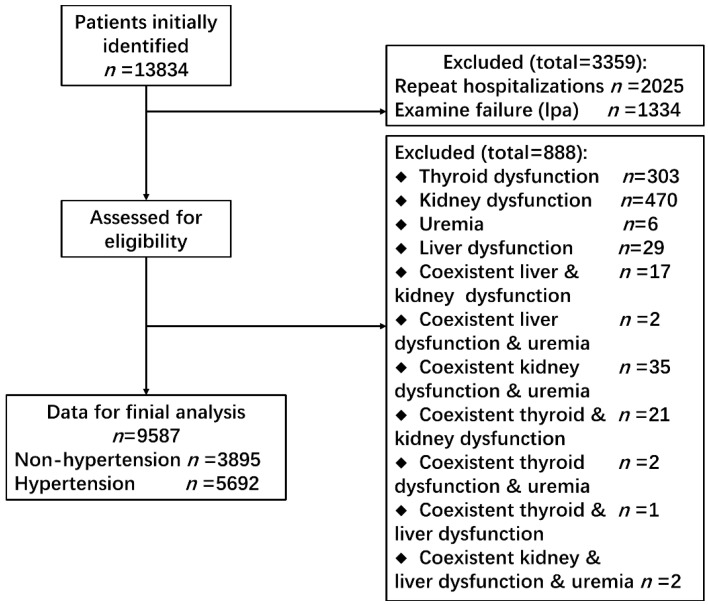
Flow diagram of patient selection.

The patient enrollment was described fully elsewhere [15]. In short, the eligible patients were hospitalized and aged 18 years or over in the department of cardiology. Exclusion criteria included: (1) patients with thyroid abnormalities; (2) patients with liver function abnormalities; (3) patients with kidney function abnormalities or uremia; (4) patients with secondary hypertension; and (5) patients with coexistence of any entities mentioned above. Details are shown in Figure 1.

### 2.3. Lab Examinations

All patients were examined in the morning after 8 hours fasting. Fasting blood was drawn to test serum uric acid (SUA), triglyceride (TG), total cholesterol (TC), low-density lipoprotein cholesterol (LDL-C), creatinine, and blood glucose according to the manufactures’ specifications. All the lab examinations were tested by Siemens 2400 biochemical analyzer. Height and weight were measured without shoes and wearing light clothes. The body mass index (BMI) was calculated as body weight (kilograms) divided by height (meters) squared.

### 2.4. Groupings, Risk factors and Their Definitions

Patients were divided into two groups according to the presence or absence of hypertension. Hypertension was diagnosed as systolic blood pressure (SBP)/diastolic blood pressure (DBP) ≥140/90 mmHg, or previously diagnosed as hypertension now under antihypertensive treatment. The normal range of SUA in Chinese was 240–420 μmol/L in both genders [16]. Current smokers were defined as those who smoked any tobacco in the previous 1 year or those who had quit within half a year. Past smokers were defined as those who had quit more than half a year earlier. Current alcohol consumers were defined as those who drank at least once a week in the previous 6 months. Past alcohol consumers were defined as those who had been abstinent in alcohol intake for >6 months prior to admission. Kidney dysfunction was defined as eGFR <60 mL/min/m^2^ calculated with the MDRD equation [17].

### 2.5. Statistical Analysis

All continuous variables were tested on their normality by using Shapiro–Wilk test. All of them did not conform to normality. Thus, we compared the continuous variables with a Rank-sum test and expressed the data as medium (inter quartile range, IQR). We compared categorical variables with the Likelihood-ratio Chi-squared test and expressed them as frequencies or percentages. The SUA in hypertension group was divided into quintiles according to the control group. The 1st SUA quintile was used as the reference. The ORs for the 2nd, 3rd, 4^th^, and 5th quintiles were reported in reference to the 1st one. The stratification of risk factors was performed dichotomously for dichotomous variables. Unconditional logistic regression was performed to estimate the association between SUA and hypertension. We fitted two hypertension models on SUA. Model 1: crude ORs were reported with no adjustment for any risk factors; model 2: fully adjusted ORs, with the adjustment for risk factors including sex, age, BMI, drinking, smoking, diabetes, creatinine, and LDL-C. The interaction between subgroups was calculated by an additive model. The variance inflation factor (VIF) was used to quantify the potential multicollinearity among covariates in the model. Sensitivity analysis was performed by excluding those patients with missing baseline body weight as it had the highest missing percentage among covariates. Estimates of odds ratios and 95% CIs were reported for risk factors. All statistical analyses were conducted using STATA 13.0 software (State Corp LP, College Station, TX, USA). A two-sided *p* < 0.05 was considered statistically significant.

## 3. Results

### 3.1. Distribution of SUA Concentrations

**Figure 2:**
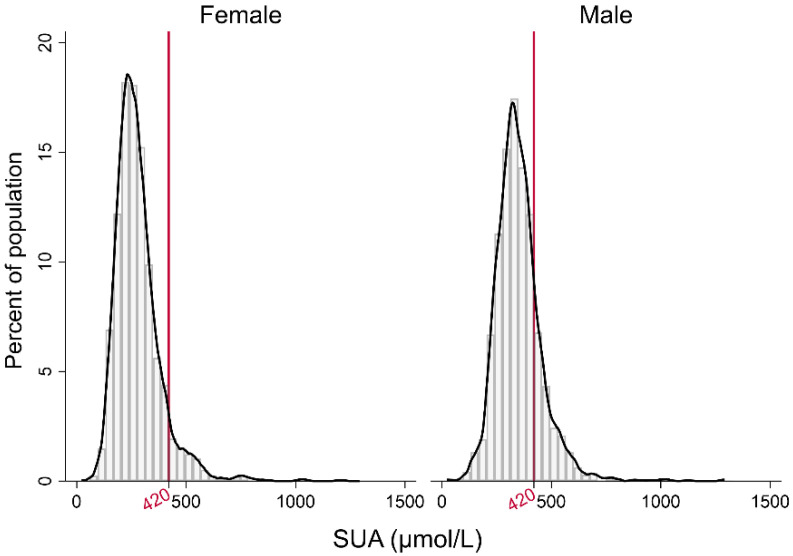
Distributions of SUA concentrations.

A total of 9587 patients were finally analyzed in the current study, where 5692 were with hypertension and 3895 were without. Both men and women had a similar distribution pattern in terms of SUA concentrations in non-hypertension controls as shown in Figure 2. On average, the SUA concentration was 259.00 (111.00) (median (IQR, inter quartile range) μmol/L in females, and 339.00 (115.00) μmol/L in males.

### 3.2. Baseline of Characteristics of Study Subjects Grouped by Hypertension

Patients with hypertension were 9 years older than those without (66 vs. 57, *p* < 0.01). The BMI in the hypertension group was higher than that in the non-hypertension group (24.09 kg/m^2^ vs. 23.83 kg/m^2^, *p* < 0.01). More than half of the individuals in both groups never smoked. Hypertensive patients had a higher past smoking proportion (10.20% vs. 6.90%, *p* < 0.01), and a lower current smoking proportion (28.20% vs. 30.30%, *p* < 0.01) than the non-hypertension group. More patients in the hypertension group drank than those in the non-hypertension group (16.90% vs. 16.70%, *p* < 0.01). Diabetes patients were more likely to be with hypertension (22.60% vs. 8.10%), and hypertension patients had a higher creatinine concentration than the non-hypertension ones (77 μmol/L vs. 72 μmol/L, *p* < 0.01). Expectedly, patients with hypertension were more likely to take antihypertensive. Details are shown in Table 1.

### 3.3. Relative Risk of SUA for Hypertension on a Continuous Scale

Table 2 showed that per 100 μmol/L higher SUA concentration was associated with multivariable-adjusted odds ratios (95% CI) of 1.25 (1.08–1.22) in males, 1.10 (1.01–1.20) in females, and 1.19 (1.13–1.24) in total. See details in Table 2.

### 3.4. Relative Risk of SUA for Hypertension on a Category Scale

**Figure 3:**
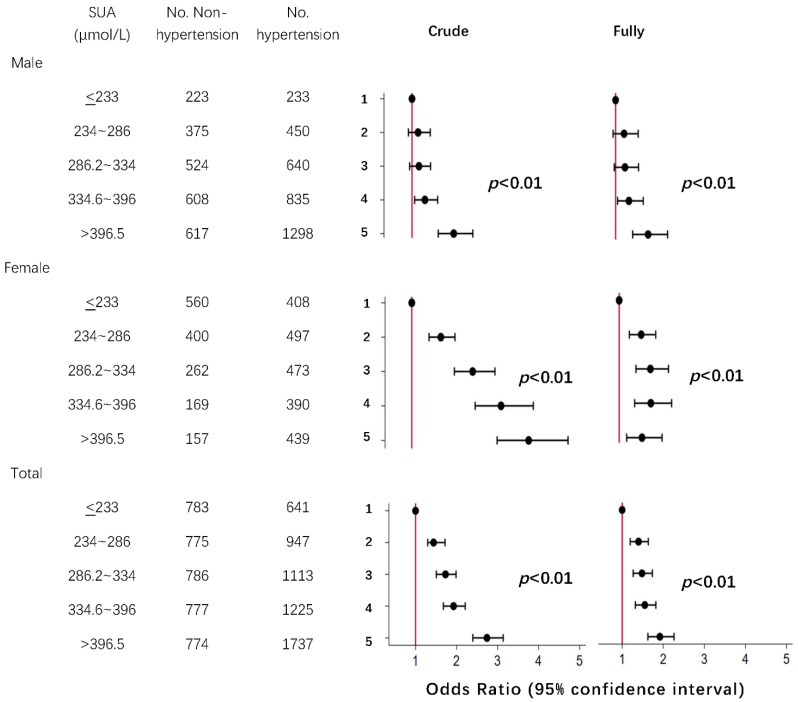
Odds ratios for hypertension based on quintiles of SUA concentrations. Notes: Horizontal axis represents OR (95% CI); vertical axis represents the quintiles; black dots represent point estimates and error bars, 95% confidence interval; crude denotes no adjustment of any risk factors; fully adjusted for age, sex, BMI, smoking, drinking, diabetes, creatinine, and LDL-C. BMI indicates body mass index and LDL-C indicates low density lipoprotein cholesterol. *p* values are tested for trend of odds ratio.

On a categorical scale, when compared with the first quintile, the multivariable-adjusted odds ratios (95% CI) were 1.40 (1.20–1.64) for the 2nd quintile, 1.48 (1.27–1.74) for the 3rd quintile, 1.55 (1.32–1.82) for the 4th quintile, and 1.92 (1.63–2.26) for the 5th quintile in total, with *p* for trend < 0.01. Figure 3 shows the strengthening associations of SUA with hypertension with its increasing quintiles. See details in Table 3.

### 3.5. Correlations of SUA with the Other Conventional Risk Factors

The correlation coefficient of SUA with age was 0.139, sex was 0.267, drinking was 0.100, smoking was 0.133, BMI was 0.116, and creatinine was 0.456, after adjusting for all the confounding factors incorporated in the final logistical model.

### 3.6. Subgroup Analysis

**Figure 4:**
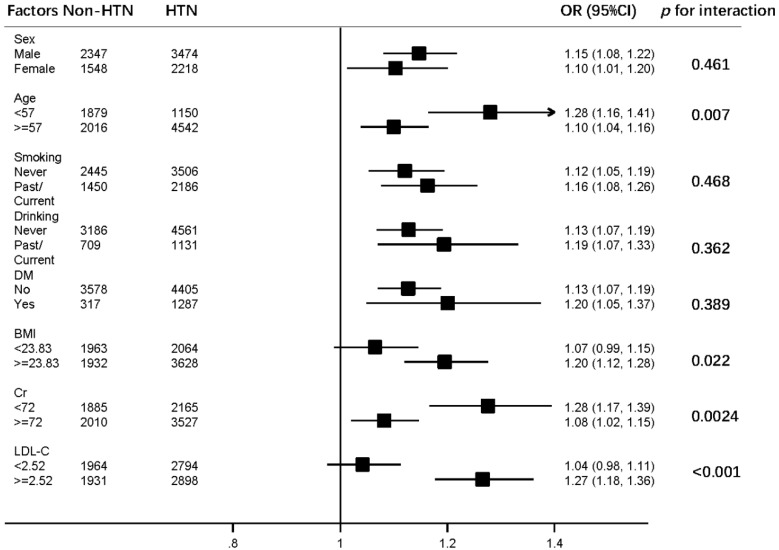
Odds ratios for hypertension per 100 μmol/L SUA increase by characteristic stratifications, dichotomously or medially. Notes: black squares represent point estimates and error bars, 95% confidence interval, adjusted for all risks mentioned in this table. *p* values for interaction in the risk of hypertension. Non-HTN indicates Non-hypertension; HTN, hypertension; DM, diabetes; BMI, body mass index; Cr, creatinine; and LDL-C, low density lipoprotein cholesterol.

The subgroup analysis confirmed the consistent associations between the SUA and hypertension although interactions existed between SUA and age, BMI, creatinine, and LDL-C as shown in Figure 4.

## 4. Discussion

The key findings in the present study were that (i) on a continuous scale, per 100 μmol/L SUA increase was associated with a 19% increased risk of hypertension in the multivariate analysis; (ii) on a categorical scale, the multivariable-adjusted associations between the SUA and hypertension were stepwise increased in a dose-response manner both in men and women; and (iii) SUA significantly interacted with age, BMI, creatinine, and LDL-C in terms of the risk of hypertension.

One study [18] found that the morbidity of hypertension was increased with higher SUA levels, even in the individual without hypertension, heart disease or diabetes; similarly, in a cohort study, asymptomatic SUA without comorbidities predicted the development of hypertension [6]; moreover, SUA also contributed to the development of hypertension from prehypertension [19]. These studies have lent support to the current study that the SUA is associated with hypertension. A meta-analysis performed on Chinese populations [7], showed that per 1 mg/dl increase in SUA was associated with an increased risk of incident hypertension (OR, 1.15; CI, 1.06–1.26), similar to our report. In the current study, we reported the fully adjusted OR (95% CI) of 1.19 (1.13–1.24) with per 100 μmol/L higher SUA levels. A previous Finnish study showed that per 81 μmol/L SUA increase (a standard deviation) was associated with the 1.43-fold increased risk of hypertension (OR, 1.43; 95% CI, 1.27–1.61) [9], which was far higher than our study (43% vs. 19%). Higher proportion of diabetes and different ethnicities perhaps resulted in this markedly strengthened SUA-hypertension association in Caucasians. A Romanian study also found that increased SUA was associated with an increased risk of hypertension (OR, 1.713; 95% CI, 1.241–2.363) [20]. The results of the two Caucasian studies are quite different from our findings, which are deserving of being further studied. 

In addition, we found broad interactions between SUA and age, BMI, creatinine or LDL-C on hypertension. These interactions were consistent with previous reports [21,22,23,24], demonstrating that management of SUA in the context of these risk factors will contribute to the prevention and control of the blood pressure.

Uric acid (UA) is a metabolite derived from the purine catabolism [25], and its homeostasis is determined by the balance between production, intestinal secretion, and renal excretion [26]. Approximately 70% of UA is easily filtered into the renal tubule, and 90% of filtered UA is reabsorbed by the S1 segment of the proximal convoluted tubule. Approximately 10% of filtered UA is finally excreted [27]. At present, there are several plausible mechanisms to explain the causally link between the SUA and hypertension: SUA can activate renin–angiotensin–aldosterone system, which can lead to retention of water and sodium [15]; SUA can promote generation of reactive oxygen species (ROS). This ROS directly reduces the bioavailability of the vasodilator nitric oxide, leading to the formation of peroxynitrite [28,29]. The increased SUA can promote oxidative stress through mitogen-activated protein kinases and reduces nicotinamide adenine dinucleotide phosphate oxidase. Oxidative stress can lead to the inhibition of cis-aconitase and acetyl-CoA synthetase, and then participates in inflammation, immune activation, vasoconstriction, intracellular angiotensin system, endothelin, thromboxane, and many other aspects [30,31,32].

The strengths and limitations should be considered in the interpretation of our study. The sample size (*n* = 9587) in the present study was large enough to evaluate association of SUA and hypertension in a Chinese hospitalized population, which helps us know the distribution shape, overall concentrations, the association, and its magnitude of SUA with hypertension. The cross-sectional study design was unavoidably open to confounding factors, which may exaggerate or attenuate the association of exposure with the main outcome. Therefore, the present findings should be considered as hypothesis-generating and should be further tested in the context of randomized trials. In addition, the material of diuretic therapy was not mentioned in the data, and we will add it in further study.

## 5. Conclusions

The SUA was associated with hypertension among a Chinese hospitalized population, and the association between the SUA and hypertension was stepwise increased in a dose-response manner. Management of the SUA could help to the prevention and control of blood pressure.

## Figures and Tables

**Table 1:** Baseline characteristics of individuals grouped by presence or absence of hypertension.

Characteristics	Non-Hypertension	Hypertension	*p* Values
*n* (%)	3895	5692	
Male *n* (%)	2347 (60.30)	3474 (61.00)	0.44
Age (IQR), year	57 (21)	66 (17)	<0.01
BMI (IQR), kg/m^2^	23.83 (3.61)	24.09 (3.63)	<0.01
Alcohol intake *n* (%)			<0.01
Never	3186 (81.80)	4561 (80.10)	
Past-drinking	59 (1.50)	171 (3.00)	
Current drinking	650 (16.70)	960 (16.90)	
Smoking *n* (%)			<0.01
Never	2445 (62.80)	3506 (61.60)	
Past smoking	269 (6.90)	579 (10.20)	
Current smoking	1181 (30.30)	1607 (28.20)	
SUA (IQR), μmol/L	310 (248, 380)	341 (279, 416.25)	<0.01
Diabetes *n* (%)	317(8.10)	1287(22.60)	<0.01
Creatinine (IQR), μmol/L	72 (61, 84)	77 (65, 91)	<0.01
LDL-C (IQR), mmol/L	2.52 (2.1, 3.08)	2.54 (2.1, 3.12)	0.36
Aspirin *n* (%)	1529 (39.30)	3393 (59.60)	<0.01
ACEI-ARB *n* (%)	1322 (33.90)	4221 (74.20)	<0.01
BBB *n* (%)	1382 (35.50)	3380 (59.40)	<0.01
CCB *n* (%)	101 (2.60)	2231 (39.20)	<0.01
Statins *n* (%)	1428 (36.70)	3183 (55.90)	<0.01

Notes: IQR indicates inter quartile range; SUA, serum uric acid; LDL-C, low density lipoprotein cholesterol; BMI, body mass index; ACEI, angiotensin converting enzyme inhibitor; ARB, angiotensin-II receptor blocker; BBB, beta blockers; and CCB, calcium channel blockers. Continuous variables are expressed as median (IQR); categorical variables are expressed as percentage. *p* values indicate comparisons among independent two groups.

**Table 2:** Odds ratios of 100 μmol/L higher SUA concentration for hypertension on a continuous scale.

Sex	Crude 0R (95% CI)	Fully 0R (95% CI)
Male	1.23(1.17–1.29)	1.25(1.08–1.22)
Female	1.52(1.42–1.63)	1.10(1.01–1.20)
Total	1.31(1.26–1.36)	1.19(1.13–1.24)

Notes: Crude denotes no adjustment of any risk factors; fully adjusted for age, sex, BMI, smoking, drinking, diabetes, creatinine, and LDL-C. OR (95%CI) indicates odds ratio (95% confidence interval); LDL-C, low density lipoprotein cholesterol; and BMI, body mass index.

**Table 3:** Odds ratios for hypertension on quintiles of SUA levels.

Quintile	Sex	OR (95% CI) ¶	OR (95% CI) ǁ	Crude or Fully *p* for Trend
Q1	Male	1(reference)	1(reference)	<0.01
Q2		1.15(0.91–1.44)	1.21(0.94–1.55)	
Q3		1.17(0.94–1.45)	1.23(0.97–1.56)	
Q4		1.31(1.06–1.62)	1.32(1.05–1.67)	
Q5		2.01(1.64–2.48)	1.79(1.41–2.26)	
Q1	Female	1(reference)	1(reference)	<0.01
Q2		1.71(1.42–2.05)	1.54(1.25–1.89)	
Q3		2.48(2.03–3.02)	1.76(1.41–2.20)	
Q4		3.17(2.54–3.95)	1.77(1.38–2.27)	
Q5		3.84(3.07–4.79)	1.56(1.18–2.04)	
Q1	Total	1(reference)	1(reference)	<0.01
Q2		1.49(1.30–1.73)	1.40(1.20–1.64)	
Q3		1.73(1.51–1.99)	1.48(1.27–1.74)	
Q4		1.93(1.68–2.21)	1.55(1.32–1.82)	
Q5		2.74(2.40–3.14)	1.92(1.63–2.26)	

Notes: ¶ denotes crude analysis; and ǁ denotes multivariable analysis for age, sex, BMI, smoking, drinking, diabetes, creatinine, and LDL-C. OR (95%CI) indicates odds ratio (95% confidence interval); LDL-C, low density lipoprotein cholesterol; and BMI, body mass index.

## Data Availability

The correspondent will be responsible for the data availability upon requested appropriately.
